# Enhancing prediction of tooth caries using significant features and multi-model classifier

**DOI:** 10.7717/peerj-cs.1631

**Published:** 2023-10-31

**Authors:** Shtwai Alsubai

**Affiliations:** Department of Computer Science, College of Computer Engineering and Sciences, Prince Sattam bin Abdulaziz University, Al-Kharj, Saudi Arabia

**Keywords:** Tooth caries detection, PCA feature engineering, Ensemble learning, Voting classifier, Chi-square, Feature extraction

## Abstract

**Background:**

Tooth decay, also known as dental caries, is a common oral health problem that requires early diagnosis and treatment to prevent further complications. It is a chronic disease that causes the gradual breakdown of the tooth’s hard tissues, primarily due to the interaction of bacteria and dietary sugars.

**Results:**

While numerous investigations have focused on addressing this issue using image-based datasets, the outcomes have revealed limitations in their effectiveness. In a novel approach, this study focuses on feature-based datasets, coupled with the strategic integration of Principle Component Analysis (PCA) and Chi-square (chi^2^) for robust feature engineering. In the proposed model, features are generated using PCA, utilizing a voting classifier ensemble consisting of Extreme Gradient Boosting (XGB), Random Forest (RF), and Extra Trees Classifier (ETC) algorithms.

**Discussion:**

Extensive experiments were conducted to compare the proposed approach with the chi^2^ features and machine learning models to evaluate its efficacy for tooth caries detection. The results showed that the proposed voting classifier using PCA features outperformed the other approaches, achieving an accuracy, precision, recall, and F1 score of 97.36%, 96.14%, 96.84%, and 96.65%, respectively.

**Conclusion:**

The study demonstrates that the utilization of feature-based datasets and PCA-based feature engineering, along with a voting classifier ensemble, significantly improves tooth caries detection accuracy compared to image-based approaches. The achieved high accuracy, precision, recall, and F1 score emphasize the potential of the proposed model for effective dental caries detection. This study provides new insights into the potential of innovative methodologies to improve dental healthcare by evaluating their effectiveness in addressing prevalent oral health issues.

## Background

Maintaining good oral health is crucial for overall well-being and enhancing one’s quality of life. Oral health encompasses the absence of throat cancer, mouth infections, sores, tooth decay, dental loss, gum problems, and related issues leading to impede activities such as chewing, speaking, smiling, biting, and overall psychosocial well-being (Samim et al., 2016). Dental caries, a common oral health problem, is caused by the interaction of oral bacteria and dietary sugars. Bacteria break down sugars into acids, which erode tooth enamel over time (Strużycka, 2014). Repeated exposure to these acids can lead to demineralization, which causes cavities, or localized damage to tooth structure. Dental caries is a chronic disease that affects the hard tissues of the teeth, including enamel, cementum, and dentin. It is caused by the interaction of oral bacteria and dietary sugars. The bacteria produce acids that demineralize the tooth enamel, leading to the formation of cavities (Kudkuli et al., 2020). Over time, the caries can progress inward, destroying the dentin and cementum.

Almost half of the global population is affected by oral diseases in one way or the other, with over 2.2 billion individuals worldwide having dental caries of a permanent nature (James et al., 2018). Dental caries, also known as tooth decay, is a condition caused by the interaction of oral bacteria and dietary sugars. Oral bacteria produce acids like lactic acid by breaking down sugars, which directly impact the tooth’s enamel layer. This acid erosion can gradually create gaps or cavities between teeth. If untreated, these cavities can cause pain, infections, and even lead to tooth loss (Pihlstrom & Tabak, 2005; Selwitz, Ismail & Pitts, 2007; Ogden et al., 2002). Dental imaging techniques can help to prevent the progression of caries lesions to more serious stages (Gomez, 2015; Metzger et al., 2022; Michou et al., 2022). Early-stage caries, also known as incipient caries, is the first stage of tooth decay. It is caused by acids produced by bacteria in the mouth when they break down sugars. These acids can demineralize the enamel, the hard outer layer of the tooth. The outer tooth layer may still be intact at this stage, making the damage difficult to see, even with some imaging techniques. The identification and description of specific areas of a tooth are facilitated by the use of named surfaces and directions that are designated according to their location. Smooth surfaces are the flat areas of a tooth’s crown, occlusal surfaces are the chewing surfaces of posterior teeth, and proximal surfaces are the sides of teeth adjacent to neighboring teeth. Each of these surface types has specific characteristics and considerations in terms of dental care and hygiene. Proximal surfaces, which are the sides of teeth adjacent to neighboring teeth, can be particularly vulnerable to caries. However, early-stage caries is a crucial time for preventive action, as it is the most easily reversible stage of tooth decay. The accuracy of diagnosis relies on the skill of the dentists and the quality of the diagnostic equipment employed (Topping & Pitts, 2009).

While dental caries are preventable and treatable, they often correlate with tooth loss and discomfort, particularly in advanced stages. Effective and timely treatment hinges on the early identification of these caries. Visual examination and probing of visible dental cavities are carried out using dental probes and handheld mirrors. These conventional techniques are valuable in identifying easily accessible yet partially concealed caries (Fejerskov, Nyvad & Kidd, 2015). Kielbassa et al. (2006) examined the existence of cavities within proximal caries lesions using varying levels of magnification. However, for hidden or inaccessible lesions, X-ray radiography plays an irreplaceable role in diagnosis. Periapical, bitewing, and panoramic X-rays are commonly utilized radiographs in clinical dental practice. Periapical and bitewing X-rays detail a specific oral area by focusing on it, while panoramic X-rays capture the entire maxillofacial region (Kaur et al., 2017). Bitewing radiography is commonly used to detect caries lesions and their depths with its high sensitivity and specificity (Schwendicke, Tzschoppe & Paris, 2015). However, it cannot provide a complete assessment of all mouth lesions in a single attempt. On the other hand, Panoramic imaging provides a single, two-dimensional image of the entire upper and lower jaws, as well as the surrounding structures, such as the teeth, gums, and bone. This allows dentists to visualize the entire oral cavity in one view, which can be helpful for diagnosing and planning treatment for a variety of dental conditions (Rushton, Horner & Worthington, 1999). Due to its cost and efficacy, panoramic imaging is a widely used and accepted radiological tool for diagnosis, clinical dental disease screening, and treatment evaluation.

Various methods have been explored for the detection of dental caries in previous studies. These include the trans-illumination method (Datta, Chaki & Modak, 2019), the international caries detection and assessment system (Sinton et al., 1997), calibrated diaphragm computed tomography (Kaur et al., 2017; Schwendicke, Tzschoppe & Paris, 2015), and quantitative light-induced fluorescence (Zantner, Martus & Kielbassa, 2006). Additionally, research has been conducted on panoramic radiology procedures to improve their performance for dental caries diagnosis through image processing techniques (Abreu, Tyndall & Ludlow, 2001). Computer-aided caries detection using X-rays has also been investigated (Neuhaus et al., 2015; Alammari et al., 2013; Tracy et al., 2011). In one study, Alammari et al. (2013) utilized the Logicon Caries Detector (LCD) with modified computer-aided design software, and the effectiveness of density analysis was confirmed as an auxiliary tool to assist dentists in detecting and organizing caries based on user feedback. Advanced dentinal diagnostic tools are utilized to detect caries, enabling dentists to diagnose dentinal cavities at a significantly earlier stage compared to traditional and conventional approaches.

In recent decades, there has been a growing interest among scientists in utilizing machine learning techniques for the detection of dental diseases. Traditionally, the evaluation of radiographs and lesion detection is performed physically and subjectively by operators or experts. However, when dealing with large volumes of image data, this task can become tedious and prone to misinterpretations. Computer-aided diagnostics (CAD) using machine learning gaining the attention of many health professionals for the rapid and precise detection of dental diseases. This study investigated the use of CAD systems for the detection of dental disease. The study made the following contributions.

- The study leverages a more robust feature-based dataset, addressing the limitations of previous research on dental caries detection.
- The feature extraction process is enhanced by using principal component analysis (PCA) and chi-square ( $\chi{^2}$) to improve data insights.
- Introducing a hybrid model combining PCA features and ensemble classifiers including Extreme Gradient Boosting (XGB), Random Forest (RF), and Extra Trees Classifier (ETC).
- A voting classifier using PCA features outperformed other methods and shows potential in improving early diagnosis and treatment outcomes for effective dental caries detection.

The rest of this article is arranged in the following order. The Related Work provides a review of the relevant literature on the current study. The Method and Materials describes the dataset, methodology, and machine learning models used in this study. The findings of the article are presented in the Results and discussed in the Discussion section. Finally, the Conclusion concludes the study and outlines future research directions.

## Related work

The combination of data mining and machine learning represents a powerful tool for addressing various challenges. Analyzing potentially large medical data manually is particularly difficult because of its extensive feature vector. Machine learning has emerged as a crucial technique in numerous application domains, including healthcare (Ahmad, Eckert & Teredesai, 2018). It has demonstrated its significance by offering precise and accurate systems for applications related to medicine, even during sensitive data handling in the medical domain (Kaissis et al., 2020). Similarly, ML models have been successfully utilized in recognizing early-stage hazards associated with conditions such as dental caries.

In a study conducted by Kang, Njimbouom & Kim (2023), a dental caries detection model was proposed. This approach involved integrating the minimum Redundancy Maximum Relevance (mRMR) and GINI index (GINI) algorithms with the Gradient Boosting Decision Tree (GBDT) classifier. By utilizing only a few clinical test features, this method aimed to save time and cost during dental caries screening. The planned method was matched to other recently recommended dental processes. Among the different classifiers, the highest classification performance was demonstrated by GBDT having a reduced feature set. It achieved precision, impressive accuracy, F1-score, and recall values of 99%, 95%, 93%, and 88% respectively. In their research, Thanh et al. (2022) put forward a Deep Learning-based system for detecting dental caries by using photos taken by a smartphone from inside the mouth. They employed four deep learning models, namely RetinaNet, YOLOv3, Faster Region Convolutional Neural Network (R-CNNs), and Single-Shot Multi-Box Detector (SSD), to identify early-stage cavities and caries lesions. This study indicated that YOLOv3 achieved the highest sensitivity value, specifically 87.4%. This suggests that YOLOv3 exhibited strong performance in accurately detecting dental caries lesions in the intraoral photos captured by smartphones.

Lian et al. (2021) offered a system based on deep learning for the detection and grouping of caries. The dataset in the study was self-annotated. The authors applied a convolutional neural network (nnU-Net) to detect caries lesions and DenseNet121 to classify the lesions based on their depths. The performance of DenseNet121 and nnU-Net models was compared with outcomes from six dentists on the test dataset using various evaluation metrics, including Dice coefficient, intersection over union (IoU), recall, accuracy, negative predictive value (NPV), precision, and F1-score. This showed that nnU-Net achieved caries lesion segmentation Dice coefficient and IoU values of 0.663 and 0.785 respectively. The recall and recall rates of nnU-Net were 0.821 and 0.986, respectively. This shows the effectiveness of the nnU-Net model in accurately segmenting caries lesions. Oztekin et al. (2023) utilized panoramic radiograph images for dental caries prediction. They employed deep learning models, specifically DenseNet-121, EfficientNet-B0, and ResNet-50. The study’s results revealed that the deep learning model ResNet-50 achieved an accuracy score of 92% in predicting dental caries.

AL-Ghamdi et al. (2022) proposed a neural search architecture network (NASNet) for tooth caries detection. They also equated the performance of NASNet against AlexNet and CNN prototypes. The results of the study indicated that the proposed NASNet model outperformed the rest of the deep learning models with respect to accuracy. It detected tooth caries with a 96.51% accuracy score, which is by far very impressive. Jader et al. (2018) steered a study using 1500 panoramic X-ray radiographs to develop a tool profile using a convolutional neural network based on mask region. The evaluation of the tool was based on precision, accuracy, recall, F1-score, and specificity as outcome metrics. The results of the investigation showed impressive performance metrics, with recall, F1-score, accuracy, precision, and specificity values of 0.84, 0.88, 0.98, 0.94, and 0.99, respectively. Kielbassa, Mueller & Gernhardt (2009) suggested that combining minimally invasive restoration with extensive remineralization can help prevent tooth decay, reduce the need for future fillings, and save money. This approach is consistent with the principles of minimal intervention dentistry. Similar to dentinal caries that have recently crossed the enamel-dentin border, such lesions necessitate specific treatment decisions. They may require either conventional restorations or a notably less invasive treatment approach, as highlighted in Kielbassa et al. (2020).

Muramatsu et al. (2021) utilized a fourfold cross-validation technique with a dataset having 100 dental radiographs of a panoramic nature to develop an object detection network. The network’s performance was evaluated based on sensitivity and accuracy for tooth detection. The study reported a sensitivity of 96.4% and an accuracy of 93.2% for tooth detection. Raith et al. (2017) employed a convolutional neural network (CNN) architecture along with the PyBrain package to identify teeth. They achieved a performance score of 0.93, indicating a high level of accuracy in accurately identifying teeth using their proposed approach. Kühnisch et al. (2022) utilized a model based on deep learning, specifically a convolutional neural network (CNN), for revealing and classification of caries. They compared the diagnostic performance of their model with expert standards in various scenarios. The outcome of the study suggested that the CNN prototype achieved an accuracy score of 92.5%.

The complete summary of the related work is shown in Table 1.

**Table 1:** Summay of the related work.

Reference	Classifiers used	Dataset used	Achieved accuracy
Kang, Njimbouom & Kim (2023)	GINI, mRMR, GBDT	Korea centers for disease control and prevention dataset, 2018	95% GBDT
Thanh et al. (2022)	R-CNNs, RetinaNet, Faster YOLOv3, and Single-Shot Multi-Box Detector (SSD)	Self made using mobile camera	87.4% sensitivity using YOLO3
Lian et al. (2021)	nnU-Net, DenseNet121	Stomatology hospital dataset	0.986, nnU-Net
Oztekin et al. (2023)	EfficientNet-B0, DenseNet-121, and ResNet-50	Firat university faculty of dentistry, dataset	92.00%, ResNet-50
AL-Ghamdi et al. (2022)	CNN, AlexNet, NASNet Model	Noor medical imaging center, dataset (kaggle)	NASNet Model 96.51%
Jader et al. (2018)	Mask R-CNN	Silva, Oliveira & Pithon (2018) dataset	98% accuracy using Mask R-CNN
Muramatsu et al. (2021)	CNN with single and multiple data inputs	Asahi university hospital, dataset	93.2%
Raith et al. (2017)	ANN, CNN with PyBrain package	Ludwig-maximilians-university munich dataset	93% CNN
Kühnisch et al. (2022)	CNN (using different amount of data *e.g.*, 25%, 50%, 75% and 100% of the dataset)	Self made, using Nikon D7100, D300, or D7200 with a Nikon Micro 105-mm lens	92.5%
Moutselos et al. (2019)	Mask RNN for object detection and DNN for segmentation	Self made	88.9% mask RNN

## Materials and Methods

**Figure 1:**
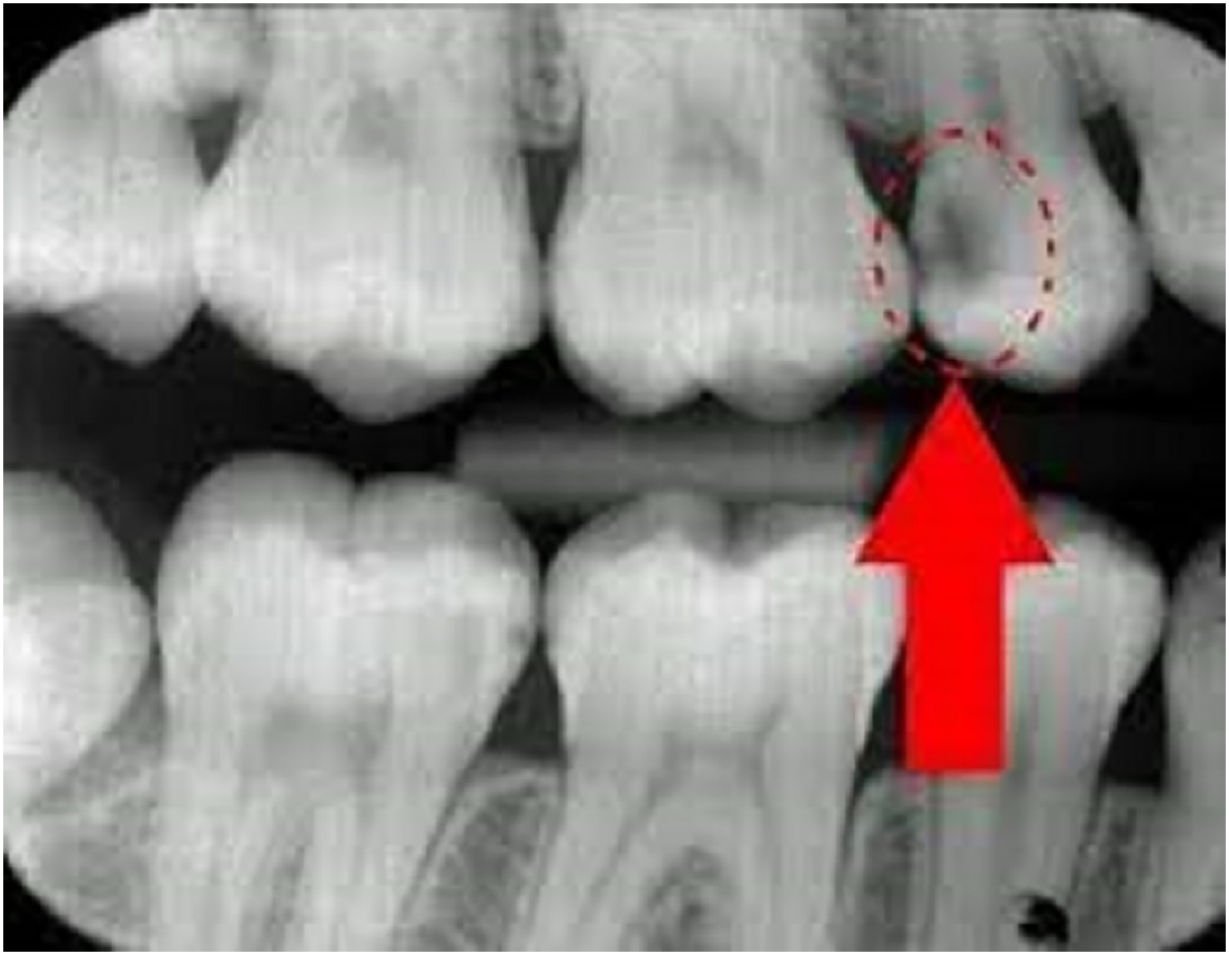
Radiograph explanation of tooth carries and normal.

This section explains the methodology and procedures employed in this study in detail. Figure 1 depicts the architecture of the suggested technique. Starting with data retrieval, the approach follows the generation of text by Synthetic Minority Over-sampling Technique (SMOTE) to balance the dataset. Feature extraction is then carried out that involves term frequency (TF), term frequency-inverse document frequency (TF-IDF), and CNN. The data is split for training and testing where the selected machine learning models are utilized for sentiment classification.

### Tooth caries dataset

The data used in this research work originates from the Oral Health Survey carried out by dentists at the university. Dentists performed oral examinations in each university to conduct this survey and to get accurate results. A total of 10,375 students participated in this survey, and a questionnaire about oral health awareness was chosen for data collection. The radiograph explanation of tooth carries and normal is shown in Fig. 1. This questionnaire uses the same attributes that were collected by Korean dentists in their research work (Kang, Njimbouom & Kim, 2023). This questionnaire comprised 43 items and a single label, covering various aspects such as place of residence, gender, age, tooth brushing frequency, snack frequency, oral care usage, oral health awareness, smoking experience, and behavior. The label for this dataset is “act_caries.” It is worth noting that since the data does not contain any personally identifiable information about patients, it does not require approval from an Institutional Review Board (IRB). For detailed descriptions of the questionnaire items, please refer to Table 2.

**Table 2:** Dataset attributes details.

Variable	Description
act_caries	It represents the presence of the dental caries (Label)
Sido_No	It shows the area of residence of the respondent of the dental examination.
Region_No	It represents the region of residence of the subject.
Gender	It represents the gender of the respondent.
Prev_caries	It represents the previous history of dental caries.
Calculus	It represents respondent have tartar build-up
Fluorosis	It represents tooth speckle
Bleeding	It show the gingival bleeding
X1	It shows the awareness of the respondent about the dental and gum oral health
X2	It show the respondent dental treatment experience in the last year
X3	It shows the respondent experience of the needing dental treatment but not receiving treatment.
X4_1	It shows the tooth brushed before breakfast
X4_2	It shows the tooth brushed after the breakfast
X4_3	It shows the tooth brushed before lunch
X4_4	It shows the tooth brushed after lunch
X4_5	It shows the tooth brushed before dinner
X4_6	It shows the tooth brushed after dinner
X4_7	It shows the tooth brushed after snack
X4_8	It shows the tooth brushed before going to sleep
X4_9	Tooth not brushed
X5_1	Frequency of the dental floss usage
X5_2	Frequency of handle floss usage
X5_3	Frequency of mouth wash usage
X5_4	Frequency of electric tooth brush usage
X5_5	It represent oral care product usage (if any)
X6	It represent the toothpaste usage
X7	It represents the fluoride tooth paste usage
X8	It represent if any sticky snack eaten today?
X9	It represent if any sticky snack eaten yesterday
X10	It represent the gum bleeding of gum pain while brushing
X11	It shows the pain or discomfort in the tooth in the last 1 year.
X12	It shows parents are smoking or not
X13	It represents any smoking experience?
X14_1	It shows that the respondent living with grandfather
X14_2	It shows that the respondent living with grandmother
X14_3	It shows that the respondent living with father
X14_4	It shows that the respondent living with stepfather
X14_5	It shows that the respondent living with mother
X14_6	It shows that the respondent living with stepmother
X14_7	It shows that the respondent living with older sister/older brother
X14_8	It shows that the respondent living with younger sister/younger brother
X14_9	It shows the not living with the above mentioned family members
X15_1	It represents the house hold economic status
X16	It represents the weekly allowance

### Proposed approach

**Figure 2:**
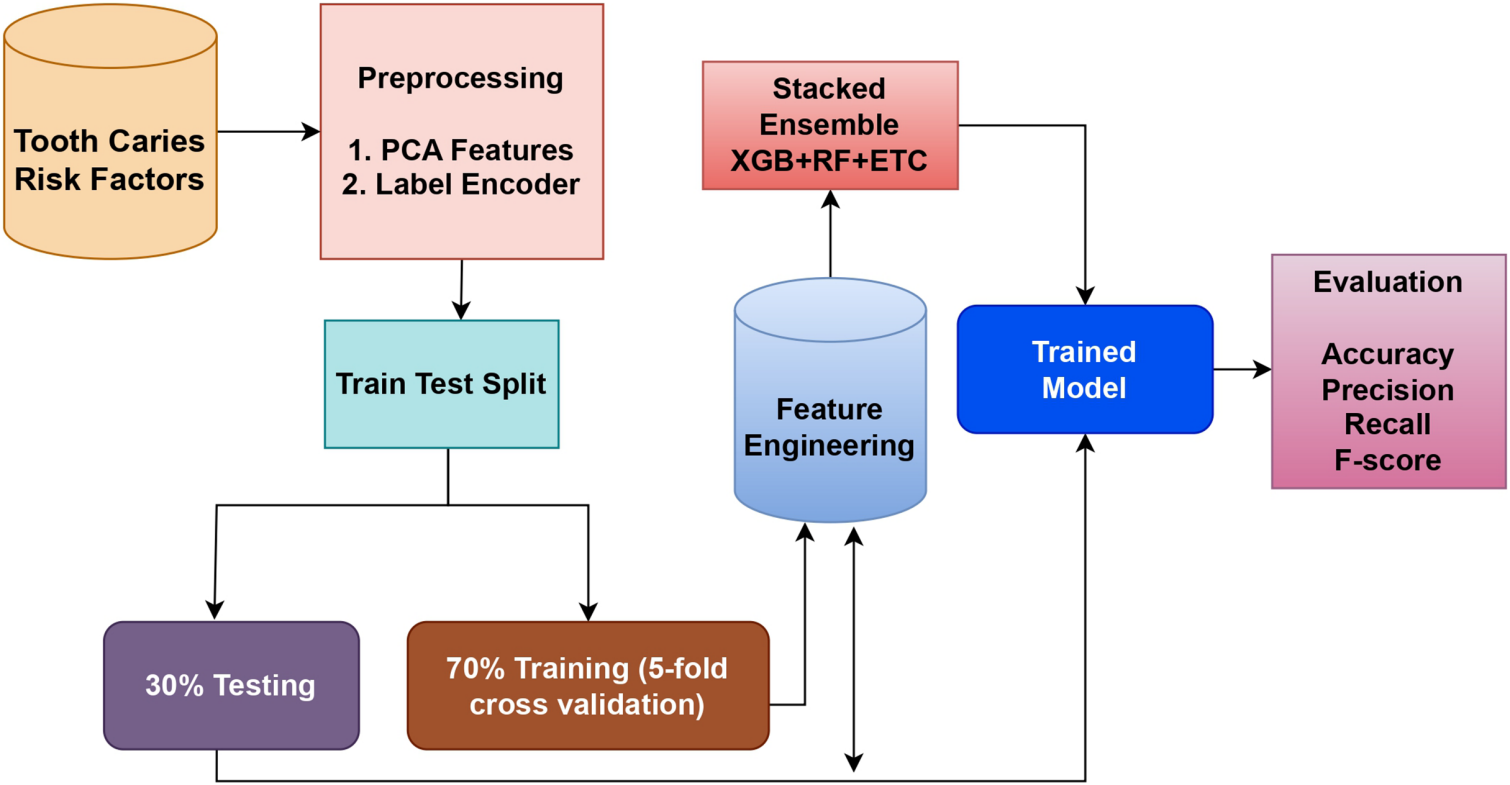
Tooth caries detection workflow methodology.

This study aimed to introduce an ML-based detection approach utilizing classifiers for more accurate prediction of tooth caries. Figure 2 depicts the design of the tooth caries detection workflow.

In this approach, the very crucial and prominent step is the feature selection. PCA and $\chi{^2}$, the two unique techniques used to select the features, are employed for feature fusion. Following this, the dataset is divided into a testing ratio of 30% and a training sets ratio of 70%. For classification purposes, an ensemble model is utilized, which combines the XGB, RF, and ETC classifiers using the soft voting criterion. Recall, accuracy, F1 score, and precision are the performance evaluation metrics of any given model or approach.

#### Proposed model XGB+RF+ETC

**Figure 3:**
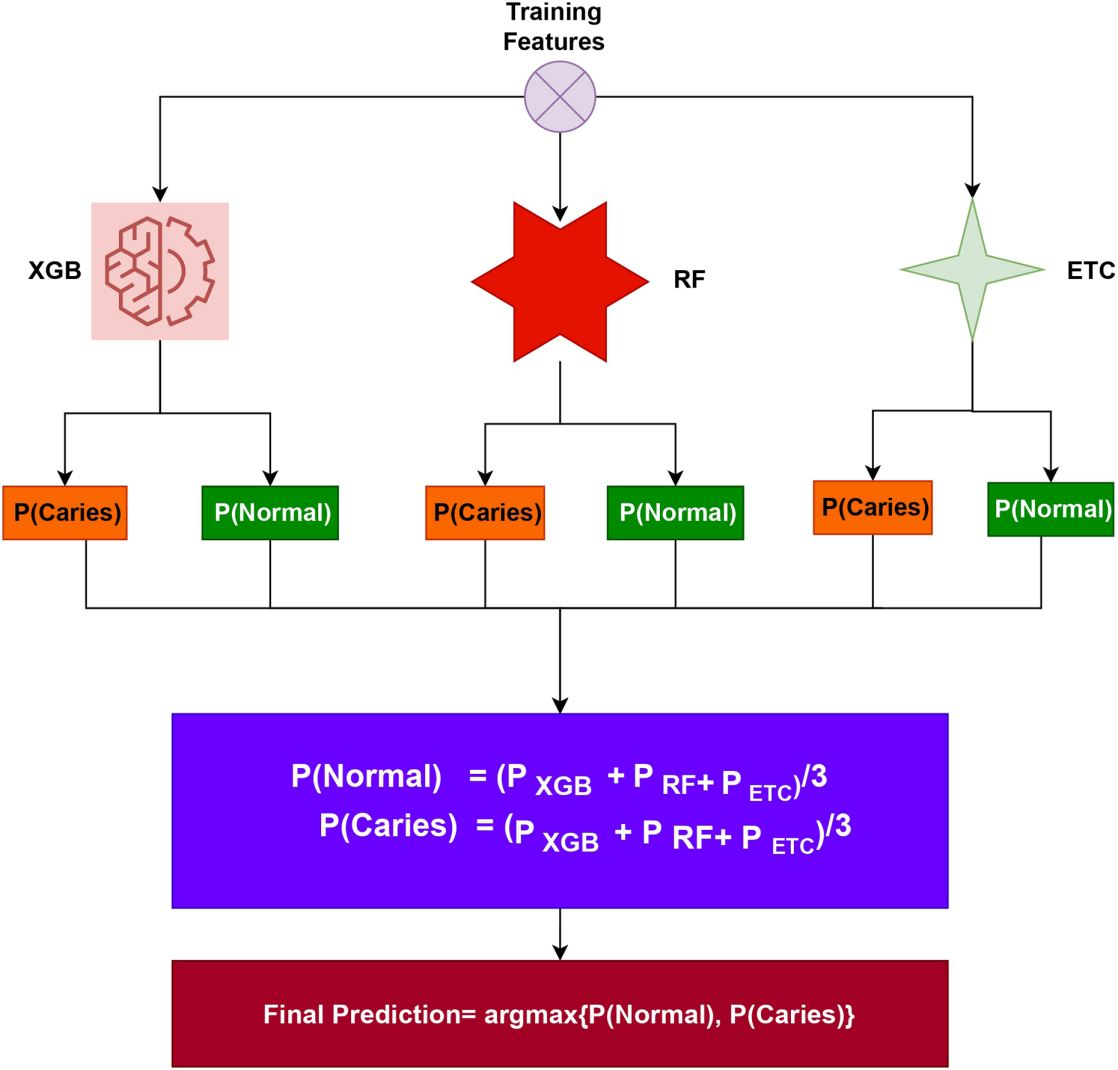
Proposed tooth caries detection framework architectural diagram.

To evaluate tooth caries detection, this study conducted experiments in three different scenarios: (i) using all features, (ii) using $\chi{^2}$-selected features, and (iii) using PCA-selected features. The dataset was then split into a 70:30 ratio, with 70% allocated to training the models and the rest of the 30% for prototype testing purposes. For the proposed tooth caries detection system, an ensemble approach called XGB+RF+ETC was utilized. Ensemble models are a powerful technique that combines predictions from multiple models to improve accuracy and robustness. Each model within the ensemble has its own strengths and weaknesses, and by combining them, the overall performance is often enhanced. In the case of tooth caries detection, this study suggests employing an ensemble learning model that integrates three popular algorithms: XGB (eXtreme Gradient Boosting), RF (Random Forest), and ETC (Extra Trees Classifier). By leveraging the strengths of these individual models and their ability to handle different aspects of the data, the ensemble approach aims to achieve better tooth caries detection results. The architecture of the proposed voting system is shown in Fig. 3.

The ensemble model has been created by combining the predictions of three different ML algorithms: XGB, RF, and ETC. The general process for constructing an ensemble model involves training multiple models on the same dataset and then merging their predictions. In the case of the XGB+RF+ETC ensemble model, each algorithm is trained individually on the dataset. During training, each model generates predicted probabilities for the different classes of the target variable. These predicted probabilities represent the likelihood of each class for a given observation. To create the final prediction for each observation, the predicted probabilities from each model are combined. One common approach is to calculate a weighted average of the predicted probabilities, where the weights assigned to each model are determined based on their performance on a validation set. Models that demonstrate better predictive performance on the validation set are assigned higher weights in the ensemble combination. By training multiple models and combining their predictions, the ensemble model aims to improve overall performance and reduce the risk of overfitting. This approach leverages the strengths of each individual model and can lead to more accurate and robust predictions for tooth caries detection. The algorithm below details the working of the recommended ensemble model, which can be expressed as:

(1) $$\hat p = argmax \left\{ \sum\limits_i^n X G {B_i},\sum\limits_i^n R {F_i},\sum\limits_i^n E T{C_i}\right\} .$$

In the case of the $\sum\nolimits_i^n X G{B_i}$, $\sum\nolimits_i^n R {F_i}$, and $\sum\nolimits_i^n E T{C_i}$ models, each of them produces prediction probabilities for each test sample. These probabilities represent the likelihood of each class for a given test case. To make a final prediction, the ensemble model utilizes the soft voting criterion. The soft voting criterion involves aggregating the probabilities generated by all three models. This aggregation process combines the predictions from XGB, RF, and ETC to arrive at a consolidated set of probabilities. Once the probabilities from all three models are combined, a final prediction is made based on these combined probabilities. For a test sample, the class having the highest probability is taken as the predicted class. By employing the soft voting criterion and combining the predictions from multiple models, the ensemble model benefits from the collective insights and strengths of XGB, RF, and ETC. This approach enhances the robustness and accuracy of the final prediction for each test case in the tooth caries detection task.

In the ensemble model, the final class prediction is determined by selecting the class with the highest average probability score among the combined predictions of the classifiers. The individual classifiers in the ensemble generate probabilities for each class in the target variable. These probabilities are combined, typically through averaging, to obtain a single probability for each class across all the classifiers. After combining the probabilities, the class having the highest average probability score is selected as the final forecast.

### Feature selection techniques

To ensure the machine learning model is trained with relevant features, feature selection methods are employed to extract and combine the particular features, resulting in a proficient feature set. The technique of feature selection plays a crucial role in achieving a well-fitted machine learning model, as each feature holds importance in relation to the object class. So, an approach has been devised to incorporate only the features having a substantial effect on the final class prediction. This approach offers numerous advantages, such as improved interpretability of learning prototypes, reduced model variations, and decreased computational costs and training time. By identifying the ideal subset of features, the complication of the system is reduced, thereby enhancing the accuracy and stability of classification. In this study, $\chi {^2}$ and PCA, are employed for this drive. Both feature selection techniques, $\chi{^2}$, and PCA aim to reduce the feature size while selecting the most relevant features. By doing so, they create a more appropriate feature set for the machine learning model.

### Chi-square ( $\chi{^2}$)

The $\chi{^2}$ feature selection method is widely utilized in machine learning, and it is also employed in the current study to identify the optimal features for model training (Narra et al., 2022). The datasets used in the experiment consist of a large number of features, which can introduce complexity in the learning process of the models. To address this, only the best features selected through the $\chi{^2}$ method are utilized to enhance the performance of the ML models. By focusing on the most informative features, the models can achieve improved performance and potentially overcome challenges associated with the large feature set. $\chi{^2}$ used the following equation to compute the score:

(2) $${X^2}(D,t,c) = \sum\nolimits_{{c_t}e[0,1]}^{} {\sum\nolimits_{{c_t}e[0,1]}^{} {{{{{(N{e_t},{e_c} - E{e_t},{e_c})}^2}} \over {E{e_t},{e_c}}}} }$$

In the given context, the variables N and E represent the observed frequency and predicted frequency, respectively. The variable ${e_t}$ is assigned a value of 1 when the text contains both “t” and “0”, and 0 otherwise. On the other hand, the variable ${e_c}$ takes the value 1 if the document belongs to the “c” class, and 0 if it belongs to any other class. A high $\chi{^2}$ score for a feature suggests that the null hypothesis (H0) of independence between the feature and the document class should be rejected. This indicates that the feature and class are interdependent, meaning that the feature has a significant impact on the frequency of occurrence within each class. In the given scenario, if the caries feature exhibits a high $\chi{^2}$ score, it implies that the feature is closely related to the document class and should be selected for training the model.

### Principal component analysis

PCA is a linear feature selection method for the identification of the most relevant features from a given dataset. It is an unsupervised method that utilizes Eigenvector scrutiny to determine the pivotal genuine features of the principal components. The principal components are linear combinations of the observed features, weighted optimally (Narra et al., 2022). Consequently, principal components are the outcome of the feature selection method of PCA, which typically has a reduced number of features compared to the genuine dataset. PCA feature selection is beneficial in various problem domains. However, it may not be preferred in cases where there is excessive multicollinearity, which refers to a high correlation among the features. In such situations, the interpretation and effectiveness of PCA may be compromised.

### Supervised models for the tooth caries detection

With the increasing popularity of machine learning models, there is a wide range of variations available in the existing literature that can deliver good classification performance. Furthermore, tools like Sci-Kit offer user-friendly functions for implementing these models. In this research, multiple machine learning classifiers are employed to classify tooth caries. The classifiers used include LR (logistic regression), DT (decision tree), RF (random forest), SGD (stochastic gradient descent), ETC (extra trees classifier), XGB (XGBoost), SVC (support vector classifier), and GNB (Gaussian naive Bayes). For comprehensiveness, each of these models is briefed as under.

#### Decision tree

The decision tree (DT) classifier is a straightforward ML algorithm that constructs relationship rules to identify and guess the particular labels (Patel & Rana, 2014; Zhang et al., 2014). It falls under the category of supervised machine learning algorithms. The decision tree starts by selecting a root node and then proceeds to traverse the tree, moving from the root node to the leaf nodes in order to make label predictions. The root node in a DT is primarily determined by two methods: Gini Index and Information Gain (IG). These methods assess the quality of potential splits in the data and choose the attribute that maximizes the information gain or minimizes the Gini index as the root node.

#### Extreme gradient boosting

EXtreme gradient boosting (XGBoost) operates similarly to the gradient boosting classifier but additionally assigns weights to each sample, like the Adaboost classifier. XGBoost is also a tree-based model that has gained a significant reputation in recent years (Zhang et al., 2014). It trains multiple weak learners, such as decision trees, in parallel, contrasting with GB, which does this chronologically. This parallel processing capability of XGBoost provides a speed boost compared to other boosting methods. XGBoost also offers L1 and L2-type regularization techniques, to control overfitting. Both the GB and the Adaboost lack this regularization technique (Freund & Schapire, 1997). Scalability is also an added benefit of XGBoost which allows it to function on distributed systems and process large datasets efficiently. In terms of the loss function, XGBoost employs the log loss function, which aids in minimizing the loss and improving accuracy. The log loss function takes into account the probability of false classifications, making it valuable for optimizing the model’s performance.

#### Logistic regression

Logistic regression (LR), is a regression adaptive method that constructs predictors using a Boolean combination of binary covariate (Sebastiani, 2002). The name “logistic regression” employs the core function deployed in this method, which is a sigmoid function. This function is characterized by an S-shaped curve, capable of turning a real-valued number into a value ranging between 0 and 1. Using the LR is suitable when we have a categorical dependent variable, making it an optimal choice for classification tasks. The logistic function can be computed as:

(3) $$y = {1 \over {(1 + {e^{( - value)}})}}$$

Overall the logistic regression can be represented as;

(4) $$y = {{{e^{{b_0} + {b_1} \times x}}} \over {1 + {e^{({b_0} + {b_1} \times x)}}}}$$

#### Stochastic gradient classifier

The stochastic gradient descent (SGD) algorithm incorporates concepts from SVM (support vector machine) and logistic regression, utilizing convex loss functions (Zadrozny & Elkan, 2002). It is a powerful classifier suitable for multi-class classification problems, employing the One-*vs*-All (OvA) approach by combining multiple binary classifiers. One of the notable advantages of SGD is its ability to handle large datasets efficiently. It achieves this by using a batch size of 1, processing only a single example per iteration. Additionally, SGD is relatively easy to understand and implement due to its basis in simple regression techniques. However, SGD does have some drawbacks. It can be quite noisy since the example chosen from the batch is random, and accurate results depend on correctly setting the hyperparameters. SGD is also highly sensitive to feature scaling, necessitating careful attention to scaling techniques for optimal performance.

#### Random forest

Random Forest (RF) is a versatile algorithm used for various tasks, including grouping, regression, and related tasks constructing multiple DTs (Gregorutti, Michel & Saint-Pierre, 2017). RF is a supervised learning algorithm that has an applicability advantage to both regressions as well as classification problems. One of the notable strengths of the RF algorithm is its high accuracy, often outperforming other existing systems. As a result, it has gained significant popularity and is widely adopted as one of the most utilized algorithms in machine learning.

#### Extra tree classifier

Extra trees classifier (ETC) is an ensemble learning method that consists of randomized trees. It aggregates multiple trees within a forest of decision trees to produce a final classification result or output (Rustam et al., 2019; Safavian & Landgrebe, 1991). The underlying concepts of both the ETC and RF are similar but contradictory in the way that DTs are constructed within a forest while in ETC, K best feature’s random samples are used for decision making. The Gini index here is employed as a mathematical criterion for selecting the best feature for splitting the data in each tree. This methodology ensures that the constructed trees in ETC are decorrelated from each other. The selection of features in ETC is based on the Gini feature importance, which determines the relevance and significance of each feature in the classification process. By considering the Gini feature importance, ETC can identify and utilize the most informative features for making decisions within each tree.

#### Support vector classifier

Support vector classifier (SVC) is a famous supervised ML prototype utilized for both regression and classification tasks (Cortes & Vapnik, 1995). It employs a hyperplane to isolate and organize the data points. It aims to locate an ideal hyperplane to maximize the distance between the hyperplane and the sample points, effectively creating a clear separation between different classes. In scenarios where the data is non-linear and cannot be separated by a linear hyperplane, SVC employs a kernel trick. This technique maps the input features into a higher-dimensional space, where the data becomes linearly separable. By transforming the data into a higher-dimensional space, SVC can effectively classify non-linear data by finding an appropriate hyperplane in the transformed feature space.

#### Gaussian naive Bayes

Gaussian naive Bayes (GNB) is a type of the naive Bayes algorithm, based on Bayes’ theorem. GNB predicts the result of an occurrence by using conditional probabilities (Ahmed et al., 2023). In GNB, if a sample is classified into k categories, denoted as k = c1, c2, $\cdots$, ck, the resulting output is assigned to one of the classes, denoted as c. The GNB function can be represented as follows, where c represents the class and d represents the sample:

(5) $$P(c|d) = (P(d|c) \times P(c))/P(d)$$

In this equation, the probability of class c given the sample d, is represented by P(c—d), P(d—c) is the probability of the sample d given class c, P(c) denotes the prior probability of class c, and P(d) represents the probability of the sample d. By calculating these probabilities, GNB predicts the most likely class for a given sample based on the principles of Bayes’ theorem.

### Evaluation parameters

To assess the performance of the ML models, four evaluation parameters are used: accuracy score, recall score, precision score, and F1 score. The accuracy score represents the percentage of correct predictions made by the prototype. To compute the accuracy, we divide the number of correct predictions by the total number of predictions made by the sample. The accuracy score ranges between 0 to 1, with one indicating a perfect prediction and 0 indicating no correct predictions. Mathematically,

(6) $$Accuracy = {{TP + TN} \over {TP + TN + FP + FN}}$$

The precision score measures the proportion of true positive predictions (correctly identified positive cases) out of all positive predictions (both true positives and false positives). It focuses on the accuracy of positive predictions.

(7) $$Precision = {{TP} \over {TP + FP}}$$

The recall score, also known as sensitivity or true positive rate, measures the proportion of true positive predictions out of all actual positive cases in the dataset. It emphasizes the model’s ability to identify positive cases.

(8) $$Recall = {{TP} \over {TP + FN}}$$

F1 score is the harmonic mean of precision and recall scores, providing a balanced evaluation metric. It is particularly useful when dealing with imbalanced datasets or when false positives and false negatives have different impacts.

(9) $$F1\;score = 2 \times {{Precision \times Recall} \over {Precision + Recall}}$$

These evaluation parameters provide a comprehensive understanding of the model’s performance, considering different aspects.

## Results

The results and outcomes of the tooth caries prediction are presented in this section. The machine learning models were implemented using Python 3.8 (Python Software Foundation, Wilmington, DE, USA) and executed within a Jupyter Notebook environment (Project Jupyter, Berkeley, CA, USA). The sci-kit learn and TensorFlow libraries (TensorFlow; Google LLC, Mountain View, CA, USA) were utilized for model development and evaluation. The experiments were conducted on a system running 64-bit Windows 10 (Microsoft Corporation, Redmond, WA, USA), featuring a 7th-generation Core i7 processor (Intel Corporation, Santa Clara, CA, USA) with a clock speed of 2.8 GHz. This information provides context regarding the hardware specifications used for the experiments. To assess the performance of the models, various evaluation metrics were employed. These include accuracy, precision, recall (sensitivity), and F1 score. To evaluate tooth caries detection, this study conducted experiments in three different scenarios: (i) using original features, (ii) using $\chi{^2}$ features, and (iii) using PCA-selected features.

### Performance of ML classifiers using original features

Experiments are performed on the original dataset features in the first phase of the tests. Table 3 shows the results of the ML models using the original features.

**Table 3:** Results of the machine learning models obtained by original from the dataset.

Model	Accuracy	Precision	Recall	F1 score
LR	85.77	90.54	91.64	90.61
DT	87.24	90.51	90.45	90.27
RF	90.65	91.35	91.75	91.21
SGD	88.59	91.37	90.88	90.66
ETC	90.08	91.35	89.35	90.12
XGB	90.51	90.95	90.89	90.93
SVC	89.45	90.34	90.34	90.62
GNB	82.38	85.44	86.12	85.99
VC(XGB+RF+ETC)	92.03	92.46	92.21	92.33

The results of the experiments indicate that the proposed ensemble model VC(XGB+RF+ETC) outclasses the individual ML classifiers in accuracy and F1-score, achieving values of 92.03% and 92.33% respectively. While the performance of all the ML classifiers is generally close to the VC(XGB+RF+ETC) model, there is a noticeable difference in accuracy and F1-score compared to the VC(XGB+RF+ETC). GNB, on the contrary, achieved the lowest accuracy score of 82.38% for the task. It demonstrates that the anticipated VC(XGB+RF+ETC) model exhibits superior performance across all evaluation metrics, including accuracy, precision, recall, and F1 score when compared to the other classifiers employed in the study.

### Results using $\chi{^2}$

Features In the second set of experiments, the ML models were trained and tested using PCA-selected features. In the third set of experiments, the same models were trained and tested using $\chi{^2}$ selected features. Table 4 presents the grouping results when $\chi{^2}$ features are used to test and train the model. The results indicate that the performance of the ML classifiers improves when features of $\chi{^2}$ are used. The proposed ensemble model VC(XGB+RF+ETC) achieves the joint highest accuracy of 91.52%, which is a 0.51% decrease from the accuracy obtained using the original features. Similarly, other machine learning models exhibit improved performance when trained on $\chi{^2}$ features.

**Table 4:** Results of the machine learning models obtained by χ^2^ features from the dataset.

Model	Accuracy	Precision	Recall	F1 score
LR	87.22	87.67	89.64	88.66
DT	88.13	88.35	88.29	88.33
RF	89.21	89.48	90.37	89.55
SGD	89.29	87.17	89.24	88.19
ETC	89.24	89.67	89.21	89.48
XGB	90.25	91.24	87.34	88.29
SVC	85.34	86.65	87.05	86.30
GNB	88.37	88.67	88.34	88.51
VC(XGB+RF+ETC)	91.52	90.25	91.61	90.47

### Results using PCA features

Table 5 presents the grouping results when the classifiers are accomplished and evaluated on PCA features. The results demonstrate that the performance of the machine learning classifiers is improved when PCA features are used.

**Table 5:** Results of the machine learning models obtained by PCA features from the dataset.

Model	Accuracy	Precision	Recall	F1 score
LR	90.24	92.82	92.71	92.80
DT	92.35	92.62	92.34	92.47
RF	94.28	95.29	95.34	95.32
SGD	91.54	93.78	93.24	93.43
ETC	95.34	95.67	95.19	95.38
XGB	94.08	94.52	94.34	94.43
SVC	93.86	94.45	93.21	93.87
GNB	90.81	91.32	90.36	90.86
VC(XGB+RF+ETC)	97.36	96.14	96.84	96.65

The proposed ensemble model VC(XGB+RF+ETC) achieves the top performance with a 97.36% accuracy score, which is a significant improvement of 5.33% compared to the accuracy obtained using the original dataset features and 5.84% compared to the Chi2 features. Additionally, the performance of GNB is also enhanced with PCA features. Moreover, SVC, LR, RF, SGD, XGB, GNB, ETC, and DT models exhibit substantial performance improvements when trained on PCA features. This suggests that PCA feature selection enhances the performance of the machine learning classifiers, including the proposed ensemble model VC(XGB+RF+ETC), in terms of accuracy.

### Comparison of machine learning models for all experiments

**Figure 4:**
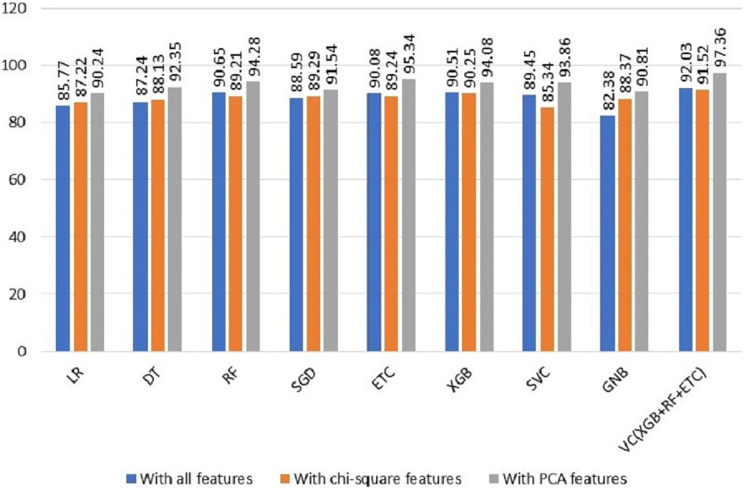
Comparison of all types of features results.

To evaluate the efficacy of the suggested system, we performed a comparative evaluation of the performance of various machine-learning models across a range of experiments. The findings revealed a noteworthy improvement in the machine learning models’ performance when utilizing the PCA features in the third experiment. Table 6 offers a comprehensive summary of the achieved outcomes by the ML models in all scenarios, facilitating a comprehensive assessment of their performance. This comparison provides a distinct understanding of the influence and advantages of incorporating PCA features in enhancing the predictive abilities of the models. The comparison of all three types of features results is shown in Fig. 4.

**Table 6:** Accuracy comparison of the machine learning models.

Model	With all features	With *X*^2^ features	With PCA features
LR	85.77	87.22	90.24
DT	87.24	88.13	92.35
RF	90.65	89.21	94.28
SGD	88.59	89.29	91.54
ETC	90.08	89.24	95.34
XGB	90.51	90.25	94.08
SVC	89.45	85.34	93.86
GNB	82.38	88.37	90.81
VC(XGB+RF+ETC)	92.03	91.52	97.36

## Discussion

Prevention and early detection play pivotal roles in dental caries management. Upholding effective oral hygiene practices, such as routine brushing with fluoride toothpaste, flossing, and utilizing antimicrobial mouthwashes, aids in plaque removal and mitigates cavity risk. Moreover, adopting a balanced diet low in sugary substances minimizes teeth’s exposure to acid-producing bacteria. Regular dental check-ups and professional cleanings are imperative for promptly identifying and addressing dental caries. Dentists are skilled at spotting early decay signs, delivering necessary interventions like fillings and offering guidance on oral hygiene practices and dietary adjustments. This research work offers a comprehensive framework for the automated detection of tooth caries. The results demonstrate the effectiveness of machine learning models for tooth caries prediction. The VC(XGB+RF+ETC) ensemble model consistently delivers high accuracy and other evaluation metrics across different feature sets. The performance improvement with PCA-selected features highlights the benefits of feature dimension reduction in enhancing model efficiency without compromising accuracy. These findings have potential implications for dental healthcare, suggesting that predictive models can play a crucial role in identifying potential tooth caries cases early, thereby facilitating timely intervention and treatment. However, further validation and testing on diverse datasets would be essential to ensure the generalizability of these models in real-world scenarios. Future work in this research will focus on combining machine and deep learning models as an ensemble to improve accuracy. Additionally, transfer learning models will be used to augment data and improve training. To further validate the results, we performed k-fold cross-validation and compared the results to state-of-the-art approaches.

### Results of the k-fold cross validation

In order to establish the models’ reliability, we utilized k-fold cross-validation. The outcomes of the 5-fold cross-validation, as depicted in Table 7, distinctly demonstrate the superiority of the suggested approach over other models in terms of recall, precision, F1 score, and accuracy. Furthermore, the proposed approach showcases a low standard deviation, underscoring its dependability and consistency. This implies that the proposed approach consistently delivers strong performance across multiple folds, instilling additional assurance in its reliability and resilience.

**Table 7:** 5-fold cross-validation results for the proposed system. The bold values show the average of all folds of the proposed model.

Model	Accuracy	Precision	Recall	F1 score
1st fold	97.52	96.13	96.61	96.12
2nd fold	97.25	96.34	96.74	96.23
3rd fold	98.64	97.67	98.98	98.81
4th fold	98.08	97.78	97.99	97.85
5th fold	96.98	96.15	96.86	96.33
**Average**	**97.89**	**96.81**	**96.23**	**96.87**

### Comparison with state-of-the-art approaches

To demonstrate and authenticate the effectiveness of the anticipated ensemble model and PCA feature approach, we conducted a performance comparison between VC(XGB+RF+ETC) and various state-of-the-art methods. The comparison results are presented in Table 8. The findings indicate that the proposed VC(XGB+RF+ETC) surpasses the performance of other modern approaches, achieving an impressive accuracy of 97.36%. This outperforms the previous best accuracy of 96.51% for tooth caries prediction, providing strong evidence of the efficacy and superiority of the proposed approach.

**Table 8:** Comparison of the proposed approach with best models from the literature. The bold values show the best performance of the proposed model among other SOTA models.

Ref	Classifier	Reported accuracy
Kang, Njimbouom & Kim (2023)	GINI, mRMR, GBDT	95.05%
Oztekin et al. (2023)	EfficientNet-B0, DenseNet-121, and ResNet-50	92.00%
AL-Ghamdi et al. (2022)	CNN, AlexNet, NASNet mmodel	96.51%
Muramatsu et al. (2021)	CNN with single and multiple data inputs	93.2%
Raith et al. (2017)	ANN, CNN with PyBrain package	93.29%
Kühnisch et al. (2022)	CNN (using different amount of data *e.g.*, 25%, 50%, 75% and 100% of the dataset)	92.50%
Moutselos et al. (2019)	Mask RNN for object detection and DNN for segmentation	88.9%
**Proposed model**	**VC(XGB+RF+ETC)**	**97.36%**

## Conclusions

This research advances the detection of dental caries by leveraging the potential of ML and CAD. Traditional methods, such as transillumination and quantitative light-induced fluorescence, are valuable but often suffer from subjectivity and manual labor. In response, this study introduces a novel framework that combines a powerful ensemble of machine learning models, including extreme gradient boosting, random forest, and extra-tree classifier, with features extracted through PCA. This work makes three key contributions: first, it presents a robust framework tailored for the accurate and efficient detection of tooth caries. Second, it comprehensively explores various machine learning models, revealing the depth of their capabilities. Third, it emphasizes the importance of feature engineering by comparing PCA and $\chi{^2}$ extraction methods. The ensemble classifier driven by PCA features achieves outstanding results in terms of accuracy, precision, recall, and F1 score reaching 97.36%, 96.14%, 96.84%, and 96.65%, respectively. This achievement not only highlights the model’s potential but also paves the way for a technology-driven transformation in healthcare. This research has the potential to revolutionize the way dental caries are detected. By reducing subjectivity, improving accuracy, and expediting diagnoses, it can help to improve oral health for people all over the world.

## Additional Information and Declarations

### Data Availability

The following information was supplied regarding data availability: The data and code are available at GitHub and Zenodo: - https://gitHub.com/MUmerSabir/ToothcariesPeerJCS. - https://github.com/shtwai/ToothcariesPeerJCS. - MUmerSabir. (2023). MUmerSabir/ToothcariesPeerJCS: ToothCariesZenodoDOI request (Zenodo). Zenodo. https://doi.org/10.5281/zenodo.8079417.
